# Experimental validation of in silico target predictions on synergistic protein targets

**DOI:** 10.1186/1758-2946-5-S1-P31

**Published:** 2013-03-22

**Authors:** Isidro Cortes-Ciriano, Alexios Koutsoukas, Olga Abian, Andreas Bender, Adrian Velazquez-Campoy

**Affiliations:** 1 Institute of Biocomputation and Physics of Complex Systems (BIFI), Unidad Asociada IQFR-CSIC-BIFI, and Department of Biochemistry and Molecular and Cellular Biology, Universidad de Zaragoza, Zaragoza, Spain; 2 Unilever Centre for Molecular Science Informatics, Chemistry Department, University of Cambridge, Cambridge CB2 1EW, UK; 3 Aragon Health Sciences Institute (I+CS), Zaragoza, Spain; 4 Fundacion ARAID, Diputacion General de Aragon, Spain

## 

Two trends are apparent in current early-stage drug discovery settings, firstly a revival of phenotypic screening strategies [1], and secondly the increasing acceptance that drugs modulate multiple targets in parallel ('multi-target drugs') [2].The work presented here combines those aspects by integrating experimental phenotypic screening for cytotoxic compounds with an experimental validation of individual protein targets modulated by the compounds. In silico target predictions for a dataset comprising cytotoxic compounds showed an enrichment of crucial enzymes for the cell cycle (such as Topoisomerase I, Bcl-X and protein kinase C alpha) and for the defense against xenobiotics (such as P-gp 1 and CYP450 enzymes). Subsequently, ten compounds from an external library (HitFinder) predicted to be active on two of the enriched targets, P-glycoprotein 1 and Topoisomerase I, were tested in vitro. Hoechst 33342 dye uptake, P-gp ATPase activity and Topoisomerase I DNA relaxation assays were able to identify two inhibitors of P-gp with IC50 values of 37 ± 5 and 28 ± 2 μM, respectively, comparable to the activity of Verapamil (12 μM). Also identified were five moderate inhibitors of Topoisomerase I inhibitors, four of which produce a synergistic effect in HeLa cell cultures in the presence of the aforesaid P-gp inhibitors (two independent samples t-test, p<0.01). Hence, this appears to be the first study work where multiple aspects of compound action - phenotypic effect as well as activity on multiple protein targets - were prospectively validated, and where partial compound synergism could be experimentally confirmed.
